# Evaluation of Functional and Radiological Outcomes of Humeral Shaft Fracture Treated With Locking Compression Plate

**DOI:** 10.7759/cureus.37197

**Published:** 2023-04-06

**Authors:** Hrushikesh Bandaru, Arun H Shanthappa

**Affiliations:** 1 Department of Orthopaedics, Sri Devaraj Urs Medical College, Kolar, IND

**Keywords:** radiological outcome, disability of the arm, locking compression plate, humerus shaft fracture, functional outcome

## Abstract

Background

The best surgical procedure for humeral shaft fractures is still plate and screw fixation. Researchers have shown that plate fixation lessens the occurrence of malunion and nonunion. This study aims to describe the functional and radiological outcomes of a humerus shaft fracture treated with a locking compression plate (LCP) using the visual analog scale (VAS) and disabilities of the arm, shoulder, and hand (DASH) scoring systems.

Method

From December 2020 to July 2022, 25 patients with humerus shaft fractures were enrolled in the prospective observational study at RL Jalappa Hospital, which is affiliated with Sri Devaraj Urs Academy of Higher Education and Research, Tamaka, Kolar. We have included closed and open type 1 fractures as per the Gustilo-Anderson classification and excluded humerus shaft fractures associated with neurovascular injury, pathological fractures, and ipsilateral upper limb long bone (radius and ulna) fractures. For a humerus shaft fracture, open reduction and internal fixation (ORIF) + LCP was done on patients who were fit for surgery, had normal test results, and were given the right kind of anesthesia. Every six weeks, every three months, and every six months, patients had regular reviews. A check X-ray was taken each time a patient attended, and we assessed them clinically and radiologically for fracture union, functional outcome, and comorbidities. The patient’s DASH and VAS ratings were assessed at the follow-up visit. The Statistical Package for the Social Sciences (SPSS) version 22.0 (IBM SPSS Statistics, Armonk, NY, USA) was used to analyze the data.

Result

The mean age of the study participants was 33 years, with a standard deviation of 8.9 years. Among the study participants, about 60% of the individuals were males. About 40% of the individuals had injuries due to motorcycle accidents, and 88% of the individuals had direct injuries. Only 12% of the individuals had disease complications. This study recorded a 100% union rate among the study samples. Among the study participants who have histories of hypertension, closed fractures have shown significant improvement according to VAS scores. Among the study participants who were males, those who presented with indirect injury, no history of fracture, right side involvement, and absence of complications showed significant improvement according to the DASH score.

Conclusion

LCP is reliable for the union of fractures in patients of any age and activity level since we can use it at all levels of the humeral shaft and can achieve 100% union when used with the right principles and osteogenic stimulus. LCPs repair humeral shaft fractures well because they can achieve good functional and radiological results and have few adverse effects.

## Introduction

Humeral shaft fractures account for about 3% of all long bone fractures [1]. Of all injuries, 63% were minor fracture patterns, while 5% were open wounds. In 18% of closed injuries, a broken humeral shaft can cause damage to the radial nerve [2]. Most of the time, they occur because of a major trauma in a child or a minor trauma in an adult. If the fracture line is situated between the brachialis muscle’s distal and proximal insertions, the injury is known as a humeral shaft fracture. Arbeitsgemeinschaft für Osteosynthesefragen (AO) defines long bone diaphyseal fractures as those that occur between the epiphyseal squares [3].

As early as 1600 BC, in Egypt, they wrote about this injury in Greek and Roman books, like the Corpus Hippocraticum [4]. This split proved difficult to treat in 20th-century writing. Campbell observed in 1924 that humerus shaft fractures were more likely to delay or result in nonunion than those of other long bones. Ghormley and Mroz confirmed a nonunion percentage of 65%. According to Caldwell’s 1933 advice, the hanging cast should be used as a movable cast because the part distal to the fracture will provide tension and pressure to correct the fractures [5].

Baseball, swimming, tennis, and volleyball depend on the kinetic chain; however, shoulder injury patterns vary by sport and position. Athletes are more likely to get hurt if they don’t follow the most important biomechanical principle for diagnosing and treating shoulder problems [6].

Younger populations have high-intensity mechanisms, while older populations have low-energy falls. There are two damage peaks in the third and sixth decades [7-9]. Between 13.4 and 14.5 per 100,000 per year for each age group, the incidence rate quickly increased to around 90 per 100,000 by the ninth decade [10]. Only 13% of humerus fractures are in the shaft, while proximal humerus fractures make up 79% of all cases [11]. Since then, the care of these fractures has changed, valuing both nonsurgical and surgical approaches. Many nonsurgical treatments were used in the years after Campbell [3,12,13], considering the surgical management of humeral shaft fractures for unique situations, such as individuals with multiple injuries, vascular damage, and other risk factors [8,14].

Since then, surgical fixation has become more common because it helps joints move and breaks heal faster [3]. The best surgical procedure is still plate and screw fixation [15]. The usual internal fixation with a plate and screw is likely to fail and cause problems [16].

The techniques of nailing and plate fixation are both being applied. They have shown that plate fixation reduces the frequency of malunion and nonunion [17]. Internal fixation reduces adult diaphyseal humerus fractures by preventing neurovascular injury. Compression-based fixation speeds healing, reduces joint stiffness, and reduces nonunion rates [18]. Plate fixation’s major drawback is soft tissue rupture, which can cause infection and nerve damage. Plating with an autologous non-vascularized fibular graft (ANVFG) has occurred. This design improves long bone gaps, atrophic nonunion biology, and biomechanical strength [19].

Nailing preserves fracture biology, reduces blood loss, speeds union, and shortens surgery. This approach has been associated with more subsequent surgeries, rotator cuff problems, and chronic shoulder pain and impingement [17,20]. Both procedures are changing rapidly, with minimally invasive plating technologies that limit soft tissue stripping and straight nails that may reduce shoulder difficulties associated with older nails.

Although there have been many meta-analyses and neither treatment method has been clearly better, plate fixation is becoming more popular [21]. It seems crucial to reassess the outcomes of this procedure, given the growing use of plating. Using the visual analog scale (VAS) and disabilities of the arm, shoulder, and hand (DASH) scoring systems, the goal of this study is to describe the functional and radiological results of a humerus shaft fracture treated with a locking compression plate (LCP).

## Materials and methods

Study design and duration

From December 2020 to July 2022, people with humerus shaft fractures took part in an observational study.

Study settings

We admitted fractured patients to RL Jalappa Hospital’s emergency room and orthopedics department, which is affiliated with the Sri Devaraj Urs Academy of Higher Education and Research, Tamaka, Kolar.

Sample size calculation

Following treatment for humeral diaphyseal fractures, Patel et al. revealed that 93.3% of the participants who had a locking compression plate had excellent or good marks on functional assessment [22]. Assuming an alpha error of 5% (95% confidence limit) and an absolute precision (d) of 10%, it was determined that 20 samples would be the bare minimum needed. The following formula was used to determine the sample size: sample size (n) = Z2*(𝑷∗𝑸)/𝒅^𝟐^, where Z = 95% confidence interval, d = absolute precision, P = proportion, and Q = 1-p. We enlarged the final sample size to include 25 patients.

Inclusion criteria

Patients above 18 years of age (skeletally mature patients) with closed humerus shaft fractures or open type 1 humerus shaft fractures (Gustilo-Anderson classification) were included.

Exclusion criteria

We excluded those with humerus shaft fractures associated with neurovascular injury, pathological fractures, and ipsilateral upper limb long bone (radius and ulna) fractures.

Sampling method

All patients were diagnosed with a fractured humerus shaft between December 2020 and July 2022 and were admitted to the emergency room and the orthopedics department of RL Jalappa Hospital (universal sampling).

Data collection procedure

Fractures were classified according to the AO classification. Until the moment of surgery, a “U”-shaped coaptation splint kept the injured limb immobile. Open reduction and internal fixation (ORIF) + LCP for a humerus shaft fracture was performed on patients who met the criteria for surgical fitness and had normal test results under appropriate anesthesia. Every six weeks, every three months, and every six months, patients had regular reviews. During each follow-up, they were given a clinical evaluation. A check X-ray was taken each time the patient attended, and they were assessed clinically and radiologically for fracture union, functional outcome, and comorbidities. The patient’s DASH and VAS ratings were assessed at the follow-up visit.

Ethical consideration

The institutional ethics committee granted its approval in terms of ethics (approval number: SDUMC/KLR/IEC/629/2020-21). After a thorough preoperative evaluation and after getting written permission from the patient, surgery was done. During the study, the researchers made sure that the participants’ privacy and anonymity were protected by only using the data for what it was meant for.

Data analysis

The Statistical Package for the Social Sciences (SPSS) version 22.0 (IBM SPSS Statistics, Armonk, NY, USA) was used to analyze the data after we imported it into Microsoft Excel (Microsoft Corporation, Redmond, WA, USA). Frequency analysis and percentage analysis were employed to characterize the data using descriptive statistics for discrete variables. For continuous variables, mean, median, and standard deviation were used. We examined discrete variables in the two groups for statistically significant differences using the chi-square test or Fisher’s exact test to characterize the data in inferential statistics. Using the independent T-test, continuous variables in the two groups were examined for statistically significant differences.

## Results

In Table 1, we detailed the study population’s basic characteristics. The study participants had a mean age of 33 years and a standard deviation of 8.9 years. About 60% of the participants who took part in the study were males. Roughly half (52%) of the study population consisted of those who had originally come from rural areas. About a third of the participants in the research also had diabetes, and almost as many had hypertension as both conditions combined.

**Table 1:** Basic characteristics of the study population *Mean and standard deviation

Variable		Frequency	Percentage
Age		33.32 ± 8.93*	
Gender	Male	15	60
	Female	10	40
Place of residence	Rural	13	52
	Urban	12	48
Hypertension	Yes	8	32
	No	17	68
Diabetes mellitus	Yes	7	28
	No	18	72

About 40% of the population was hurt in a motorcycle-related accident. Roughly 88% were hurt directly. Only around 8% showed evidence of past history of injury in the same arm. Of the participants in the study, 76% showed signs of right-sided arm involvement. About 64% were classified as having a closed fracture, while the rest were classified as having a type 1 open fracture. There was a complication rate of roughly 12% among the studied participants. In Table 2, we described the characteristics of fractures among the study population.

**Table 2:** Characteristics of fractures among the study population

Variable		Frequency	Percentage
Mode of Injury	Assault	3	12
	Fall from height	6	24
	Motorcycle accident	10	40
	Motor vehicle accident	6	24
Mechanism of injury	Direct	22	88
	Indirect	3	12
Past history of injury in the same arm	Yes	2	8
	No	23	92
Site of involvement	Left	6	24
	Right	19	76
Diagnosis	Closed fracture	16	64
	Type 1 open fracture	9	36
Complication	Yes	3	12
	No	22	88

Of the study population, 68% experienced localized swelling at the injury site. About 76% showed signs of shortening in the injured area. Crepitus was found in approximately 76% of the injured participants. In Table 3, we described the reported symptoms among the study population.

**Table 3:** Reported symptoms among the study population

Variable		Frequency	Percentage
Swelling	Yes	17	68
	No	8	32
Shortening	Yes	19	76
	No	6	24
Crepitus	Yes	19	76
	No	6	24

Participants’ reports of pain decreased significantly between week 6 and month 6 of the research. Participants’ DASH scores dropped dramatically between the six-week and six-month points of the trial. In Table 4, we described the assessment of study participants with VAS and DASH scores at six weeks, three months, and six months.

**Table 4:** Assessment of VAS and DASH scores at six weeks, three months, and six months VAS: visual analog scale, DASH: disabilities of the arm, shoulder, and hand

Scale and period of assessment	Mean	Median	Mode	Standard deviation
VAS score at six weeks	7.080	7	6	1.288
VAS score at three months	4.160	4	4	0.850
VAS score at six months	2.520	2	2	0.963
DASH score at six weeks	55.240	58	58	10.721
DASH score at three months	31.200	33	33	9.097
DASH score at six months	11.880	9	9	4.781

The average VAS score difference between the score at six weeks and the score at six months for those with and without hypertension is 4.18 and 5.38, respectively. According to an independent T-test, the difference between these means is statistically significant (P = 0.016). According to the VAS score, participants with a history of hypertension have shown significant improvement. The mean VAS score difference between the sixth week and the sixth month is 5.06 for closed fractures and 3.67 for type 1 open fractures, according to the type of fracture diagnosis. According to an independent T-test, the difference between these means is statistically significant (P = 0.003). According to the VAS score, fracture healing has improved significantly among the study participants. In Table 5, we described the association between basic characteristics and VAS score measured at six weeks and six months.

**Table 5:** Association between basic characteristics and VAS score measured at six weeks and six months VAS: visual analog scale

Variable		Mean difference of VAS score between six weeks and six months	Mean difference of difference	P value
Gender	Female	4.400	-0.267	0.595
	Male	4.670		
Place of residence	Rural	4.380	-0.365	0.456
	Urban	4.750		
Type of injury	Direct	4.450	-0.879	0.239
	Indirect	5.330		
Past history of similar illness	Past history present	5.000	-0.478	0.597
	No history	4.520		
Hypertension	Hypertensive	5.380	-1.199	0.016
	Not hypertensive	4.180		
Side involvement	Left-side involvement	3.830	-0.956	0.087
	Right-side involvement	4.790		
Swelling	Swelling present	4.530	0.096	0.856
	No swelling	4.630		
Shortening	Shortening present	4.470	0.360	0.531
	No shortening	4.830		
Crepitus	Crepitus present	4.630	-0.298	0.572
	No crepitus	4.330		
Diabetes	Diabetes present	5.140	-0.810	0.130
	Nondiabetic	4.330		
Type of fracture	Closed fracture	5.060	1.396	0.003
	Type 1 open fracture	3.670		
Complication	Complication present	4.670	-0.121	0.873
	No complication	4.550		

The mean difference in DASH scores between the sixth week and the sixth month for males and females is 45.60 and 40.00, respectively. According to an independent T-test, the difference between these averages is statistically significant (P = 0.042). Among male study participants, the DASH score shows a significant improvement. The mean difference in DASH scores between the sixth week and the sixth month among the study participants with direct and indirect damage mechanisms is 42.05 and 53, respectively. According to an independent T-test, the difference between these means is statistically significant (P = 0.008). Among study participants with indirect injuries, the DASH score shows a significant improvement. The mean difference between the DASH score in the sixth week and the score in the sixth month for study participants with and without a history of a comparable disease is 44.26 and 33.00, respectively. According to an independent T-test, the difference between these means is statistically significant (P = 0.026). According to the DASH score, there has been a marked improvement among research participants with no comparable experience. The mean difference in DASH scores between the sixth week and sixth month for participants with left and right involvement is 36 and 45.68, respectively. According to an independent T-test, there is a statistically significant difference between these means (P = 0.002). According to the DASH score, those with right-side involvement have shown significant improvement. According to the existence or lack of complications, the mean difference between the DASH score at six weeks and at six months is 44.77 and 33.00, respectively. According to the independent T-test, the difference between these means is statistically significant. In Table 6, we described the association between the basic characteristics and the DASH score measured at six weeks and six months.

**Table 6:** Association between basic characteristics and DASH score measured at six weeks and six months DASH: disabilities of the arm, shoulder, and hand

Variable		Mean difference of DASH score between six weeks and six months	Mean difference of difference	P value
Gender	Female	40.000	-5.600	0.042
	Male	45.600		
Place of residence	Rural	41.850	-3.154	0.272
	Urban	45.000		
Type of injury	Direct	42.050	-10.955	0.008
	Indirect	53.000		
Past history of similar illness	Past history present	33.000	11.261	0.026
	No history	44.260		
Hypertension	Hypertensive	41.500	2.735	0.376
	Not hypertensive	44.240		
Side involvement	Left-side involvement	36.000	-9.684	0.002
	Right-side involvement	45.680		
Swelling	Swelling present	43.350	0.022	0.994
	No swelling	43.380		
Shortening	Shortening present	44.370	-4.202	0.209
	No shortening	40.170		
Crepitus	Crepitus present	44.840	-6.175	0.091
	No crepitus	38.670		
Diabetes	Diabetes present	42.140	1.690	0.600
	Nondiabetic	43.830		
Type of fracture	Closed fracture	45.810	6.813	0.017
	Type 1 open fracture	39.000		
Complication	Complication present	33.000	11.773	0.004
	No complication	44.770		

## Discussion

Our research examined the functional and radiological outcomes of managing fractures with LCP osteosynthesis. LCP was used to treat nearly 25 patients with humerus shaft fractures along with ORIF.

The participants in the current study were on average 33 years old, with a nine-year standard deviation. The study carried out by Kumar et al. [23] had study participants who were on average 41 years old. In a similar vein, the study of Jiang et al. [24] found that the median age of study participants was 42.9 years. In contrast to our study, Capitani et al. [25] had study participants who were on average 55.45 years old. Because we carried the investigations out in various contexts, there was a variance in the mean age between the studies.

More than half (60%) of the samples in the current study were males, as opposed to the study by Kumar et al. [23], where 80% of the samples were males. The study conducted by Jiang et al. [24] revealed a similar percentage of males (66.7%) to ours. The study by Boben et al. [26] had a male prevalence of almost 84%, in contrast to our findings.

In the current study, about 64% of the population reported injuries from automobile accidents. The study by Jiang et al. [24] found that, in contrast to our study, over 81% of people experience traffic accidents. Similar to our study, Boben et al. [26] found that 70% of those who took part in their study had been in car accidents. In the prospective study by Capitani et al. [25], only 29% of the study population said that they had been in a car accident, which differs from the results of our study. Because there are more motor vehicles on the road, traffic accidents are the most frequent reason for injuries.

Approximately 76% of the participants in the current study experienced right-side injuries, compared to 46% of those in the study by Kumar et al. [23]. Of the participants of the study by Jiang et al. [24], which is comparable to ours, 62% had involvement from the right side. In contrast to our study, almost 54% have right-side involvement in the report by Boben et al. [26] and 44% have right-side involvement in the prospective study carried out by Capitani et al. [25]. Because most people use their right side as their primary side of utilization, injuries to the right side are the most common.

About 64% of the participants in the current study had closed fractures, whereas the rest had type 1 open fractures. The study carried out by Jiang et al. [24] found a similar percentage of closed fractures (81%), as did our investigation. In contrast to our study, almost 84% presented with a closed type of fracture in the study conducted by Boben et al. [26]. This selection of participants is based on the fractures since closed-type fractures are more common than open-type fractures.

The DASH questionnaire gave a clear picture of how the patients felt about their function. The samples’ respective mean DASH scores after six weeks, three months, and six months were 55.24 ± 10.721, 31.20 ± 9.097, and 11.88 ± 4.781, respectively. Between the durations of six weeks and six months, there is a significant decline in DASH scores among the study participants. In contrast to our study, the study by Gowda et al. [27] found that the mean DASH scores of the samples at six weeks, three months, and six months were 25.55 ± 4.41, 16.75 ± 4.07, and 6.05 ± 3.47, respectively. Similar to this, it was noticed in the research conducted by Akbar Ali et al. [28] that the mean quick DASH scores of the samples at eight weeks, three months, and six months were 30.51 ± 6.12, 15.05 ± 4.67, and 3.57 ± 5.21, respectively. Similar findings were made in the literature by Malhan et al. [29], where the samples’ mean DASH scores at three months and six months were 35.1 and 8.9, respectively. The factor contributing to a poor or moderate DASH score in the current investigation was issues with the other upper limb. We also discovered that although participants experienced some pain in the recovered upper limb, it was not too bad, and they could still go about their everyday lives. Early limb mobilization and partial physical treatment are keys to functional recovery. An immediate boost in function followed an operation’s more predictable alignment and immediate stabilization.

There are no cases of malunion, delayed union, or nonunion among the study participants in the current study. The study by Govindasamy et al. [30] came to the same conclusion: there were no delayed unions or nonunion. However, in their study, Boben et al. [26] noted a difference after six months; there was a union rate of nearly 97% and a nonunion rate of 3%. Also, in the study conducted by Kumar et al. [23], almost 92% had union and 8% had delayed union.

Better postoperative outcomes require strict AO fixation, careful asepsis, patient education, and a well-planned rehabilitation program. These LCP fixing protocols for humeral shaft fractures will improve patient satisfaction and reduce sequelae.

Limitation

An experimental investigation with a randomized controlled trial might produce better associations because this is an observational study. This study’s results cannot be compared with those of other methods because there was no control group and only a limited percentage of patients were involved. During follow-up, complications, including infections and nerve damage, could be assessed, but the follow-up time frame is brief but could be increased to more than a year. The study can include other variables, including the time needed for the fracture to heal, the duration of the technique, the duration of stay after surgery, and movements.

Recommendation

To identify significant changes, many patients must take part in a long-term, triple-blind, randomized controlled trial. This trial should be multicentric and evaluate diverse methods to better assess functional and radiological outcomes, superficial and deep infection, nerve injury, and range of motion after surgery. We need biomechanical stability research to evaluate fixation techniques for therapeutic purposes.

## Conclusions

Almost two-thirds of the individuals in this study had closed fractures, whereas the remaining participants had type 1 open fractures. Between six weeks and six months, there is a substantial functional improvement among the research participants. In the current study, there are no cases of malunion, delayed union, or nonunion among the participants. Based on the findings, we recommend LCP as a reliable method for humeral shaft fractures because it can achieve good functional and radiological results.
